# Single-cell sequencing reveals the reproductive variations between primiparous and multiparous Hu ewes

**DOI:** 10.1186/s40104-023-00941-1

**Published:** 2023-11-14

**Authors:** Ting Ge, Yifan Wen, Bo Li, Xiaoyu Huang, Shaohua Jiang, Enping Zhang

**Affiliations:** Key Laboratory of Animal Genetics, Breeding and Reproduction of Shaanxi Province, College of Animal Science and Technology, Northwest A&F University, Yangling, 712100 China

**Keywords:** Granulosa cells, Hu sheep, Lambing number, Ovarian somatic cells, Single-cell RNA sequencing

## Abstract

**Background:**

In the modern sheep production systems, the reproductive performance of ewes determines the economic profitability of farming. Revealing the genetic mechanisms underlying differences in the litter size is important for the selection and breeding of highly prolific ewes. Hu sheep, a high-quality Chinese sheep breed, is known for its high fecundity and is often used as a model to study prolificacy traits. In the current study, animals were divided into two groups according to their delivery rates in three consecutive lambing seasons (namely, the high and low reproductive groups with ≥ 3 lambs and one lamb per season, *n *= 3, respectively). The ewes were slaughtered within 12 h of estrus, and unilateral ovarian tissues were collected and analyzed by 10× Genomics single-cell RNA sequencing.

**Results:**

A total of 5 types of somatic cells were identified and corresponding expression profiles were mapped in the ovaries of each group. Noticeably, the differences in the ovary somatic cell expression profiles between the high and low reproductive groups were mainly clustered in the granulosa cells. Furthermore, four granulosa cell subtypes were identified. GeneSwitches analysis revealed that the abundance of *JPH1* expression and the reduction of *LOC101112291* expression could lead to different evolutionary directions of the granulosa cells. Additionally, the expression levels of *FTH1* and *FTL* in mural granulosa cells of the highly reproductive group were significantly higher. These genes inhibit necroptosis and ferroptosis of mural granulosa cells, which helps prevent follicular atresia.

**Conclusions:**

This study provides insights into the molecular mechanisms underlying the high fecundity of Hu sheep. The differences in gene expression profiles, particularly in the granulosa cells, suggest that these cells play a critical role in female prolificacy. The findings also highlight the importance of genes such as *JPH1*, *LOC101112291*, *FTH1*, and *FTL* in regulating granulosa cell function and follicular development.

**Supplementary Information:**

The online version contains supplementary material available at 10.1186/s40104-023-00941-1.

## Background

Small ruminants, particularly native breed kinds, play a significant role to the livelihoods of a considerable part of human population from socio-economic aspects [1–3]. Thus, combined trials with emphasis on administration and genetic progress to improve animal outputs are of decisive significance [4–6]. Economical and biological efficiency of sheep production enterprises generally improves by increasing productivity and reproductive performance of ewes [7–11]. Hu sheep is a first-class protected local livestock breed in China and a world-renowned multiparous sheep breed. It has early sexual maturity, four seasons of estrus, two or three litters a year, and an average lambing rate of 277.4% [12]. Hu sheep are currently bred on a large scale in China's mutton sheep production system, and the litter size of ewes clearly impacts the economic efficiency.

The ovary, a critical reproductive organ consisting of follicles at several different developmental stages. The number of lambs produced is an important indicator of sheep fertility. It is also a complex quantitative trait regulated by genetic, epigenetic, and hormonal factors with a heritability of 0.03–0.10 [13]. The number of lambs produced by each ewe is influenced by the number of ovulations, and the ovulation can be genetically regulated by a single main effector gene or some micro-effector polygenes [14, 15], such as *BMPRIB* [16], *BMP15* [17], and *GDF9* [18].

The ovary is a heterogeneous organ co-regulated by multiple cells, which determines the complexity of ovarian function. Follicular development is a highly coordinated process in sheep. Follicle cyclic recruitment, spatial displacement, follicle atresia, and ovulation are implicated events resulting from the release of molecular signals by somatic cells. Cells have different functions in the specific biological cycle of the ovary and contribute to the maturation of follicles [19]. Previous studies have focused on the impact of follicles (including granulosa cells and oocytes) on ovarian function [20, 21]. There is a growing recognition of the functional role of ovarian somatic cells (endothelial cells, stromal cells, perivascular cells and immune cells) in follicular development [22, 23]. However, few studies have been conducted to investigate the effect of different cells in the ovary on reproductive performance in sheep. Therefore, establishing a functional analysis based on different ovarian cells and their specific physiological roles is important to explore the ovarian function and elucidate the mechanisms of differences in lambing number.

With the development of sequencing technologies, single-cell RNA sequencing (scRNA-seq) technology has been employed to detect the expression profiles of different tissue cells. Thousands of single-cells from a single biopsy can be analyzed by introducing unique molecular identifiers (UMI) in droplet-based protocols, reducing amplification errors and facilitating the detection of small populations of cells whose transcriptional programs are often not detected using bulk RNA sequencing [24]. ScRNA-seq technology revealed that at distinct developing stages of cells, the corresponding cell markers are different [25–27]. In addition, using this technology, studies have explored the different cellular functions and developmental trajectories of the ovary in humans and some model animals [28–30]. In sheep, this technique is currently being used to investigate sperm-related functions in males [31–33]. Whereas, there are few studies focus on ovary function of domestic animals with scRNA-seq. In the current study, this technique was conducted to explore the cellular mechanisms underlying differences in lambing number in Hu sheep.

In sheep breeds with high fecundity performance, five main causal genes control ovulation and lambing numbers [34]. However, except for the *FecB* locus in the *BMPR-1B* gene, all other loci are not associated with high fecundity traits in Hu sheep [34]. There is still a gap between the specific gene regulatory networks and lambing number. In this study, we utilized the 10× Genomics scRNA-seq technology to analyze ovarian tissue and explore the molecular mechanisms underlying high fecundity in Hu ewe, which provide new targets for molecular breeding and theoretical basis for further studies.

## Materials and methods

### Ethical statement

The present study was approved by the Animal Care and Use Committee of Northwest A&F University, China (Approval No. DK2021113). All methods and experimentations were performed in accordance with the relevant guidelines and regulations.

### Sheep management

The 6 estrus Hu ewes (average age: 3.6 years) were divided into 2 groups based on litter size from 3 consecutive parities. The highly reproductive group (HLS, *n* = 3, body weight: 40.16 ± 1.19 kg) comprised sheep with a litter size of  ≥ 3, while the lowly reproductive group (LLS, *n* = 3, body weight: 42.01 ± 0.31 kg) consisted of 3 sheep with a litter size of = 1. Sheep weight and production records are shown in Table S1(Additional file 1).

The 6 randomly selected ewes were slaughtered within 12 h of estrus. The ram test was used to determine the estrous status. Venous blood samples were collected before slaughter for testing blood biochemical and hormone levels. Unilateral ovarian tissues were collected, placed in a protective solution (MACS® Tissue Storage Solution, Miltenyi, Bergisch Gladbach, Germany), stored at 4 °C, and analyzed by scRNA-seq.

### Blood biochemical and hormone determination

Blood biochemical indicators were measured with an automatic biochemical analyzer (BK-280, Biobase, Shandong, China). Blood hormone concentration was tested according to the instructions of the estradiol ELISA kit (Kexing, Shanghai, China), luteinizing hormone ELISA kit (Kexing, Shanghai, China), follicle-stimulating hormone ELISA kit (Kexing, Shanghai, China), testosterone ELISA kit (Kexing, Shanghai, China) and progesterone ELISA kit (Kexing, Shanghai, China) (Table S2).

### ScRNA-seq

#### Sample preparation and library construction

The entire unilateral ovaries of Hu ewe were cut up, digested with collagenase 1 for 30 min and trypsin for 10 min, sieved, centrifuged, and lysed for cell counting. ScRNA-seq libraries were prepared with Chromium Single Cell 3' Reagent v3 Kits (10× Genomics, Pleasanton, California, USA) according to the manufacturer's protocol. In briefly, single-cell suspensions were loaded on the Chromium Single Cell Controller Instrument (10× Genomics, Pleasanton, California, USA) to generate single-cell GEMs. After generating GEMs, full-length cDNA was obtained through reverse transcription reactions engaged with barcoded, then disruption of emulsions by using the recovery agent and cDNA clean-up with DynaBeads Myone Silane Beads (Thermo Fisher Scientific, Waltham, Massachusetts, USA). cDNA was amplified by polymerase chain reaction (PCR). The amplified cDNA was fragmented, end-repaired, A-tailed, index adaptor-ligated, and library amplification. These libraries were sequenced on the MGISEQ-T7 platform (MGI Tech, Shenzhen, China).

#### Data preprocessing

The Cell Ranger software pipeline (v5.0.0) provided by 10× Genomics was used to demultiplex cellular barcodes, and reads were mapped to the genome and transcriptome using the STAR aligner and down-sample reads as required to generate normalized aggregate data across samples, producing a matrix of gene counts versus cells. The UMI count matrix was processed using the R package Seurat (v3.1.1) [35]. To remove low-quality cells and multiplet captures, a major concern in microdroplet-based experiments, a criterion was applied, including to filtering out cells with gene numbers less than 200, UMI less than 1,000, and log10GenesPerUMI less than 0.7. We discarded low-quality cells where > 10% of the counts belonged to mitochondrial genes, and > 5% of them belonged to hemoglobin genes. The DoubletFinder package (v2.0.2) [36] was applied to identify potential doublet. After applying these QC criteria, 41,150 single-cells were included in downstream analyses. Library size normalization was performed with the NormalizeData function in Seurat [35] to obtain the normalized count. The global-scaling normalization method “LogNormalize” normalized the gene expression measurements for each cell by the total expression and multiplied by a scaling factor (10,000 by default). The results were log-transformed.

Top variable genes across single-cells were identified using the method described by Macosko et al. [37]. The most variable genes were selected using the FindVariableGenes function (mean.function = FastExpMean, dispersion.function = FastLogVMR) in Seurat [35]. Principal component analysis (PCA) was performed to reduce the dimensionality with the RunPCA function in Seurat [35]. Graph-based clustering was performed to cluster cells according to their gene expression profiles using the FindClusters function in Seurat [35]. Cells were visualized using a 2-dimensional Uniform Manifold Approximation and Projection (UMAP) algorithm with the RunUMAP function in Seurat [35]. The FindAllMarkers function (test.use = presto) was used in Seurat [35] to identify marker genes of each cluster. For a given cluster, FindAllMarkers identified positive markers compared with all other cells.

Differentially expressed genes (DEGs) were identified using the FindMarkers function (test.use = presto) in Seurat. *P* value < 0.05 and |log_2_foldchange|> 0.58 were set as the threshold for significantly differential expression. Gene Ontology (GO) enrichment and Kyoto Encyclopaedia of Genes and Genomes (KEGG) pathway enrichment analysis of DEGs were performed using R based on the hypergeometric distribution.

#### Pseudotime analysis

Pseudotime analysis was done with the Monocle2 package [38]. The raw count was converted from the Seurat object into the CellDataSet object with the import CellDataSet function in Monocle. The differentialGeneTest function of the Monocle2 package was used to select ordering genes (qval < 0.01), which were informative in the ordering of cells along the pseudotime trajectory. The dimensional reduction clustering analysis was performed with the reduce dimension function, followed by trajectory inference with the order. The cell function was done using default parameters. Gene expression was plotted with the plot_genes_in_pseudotime function to track changes over pseudo-time.

#### GeneSwitches analysis

GeneSwitches (v0.1.0) [39] was used to discover the sequence of gene expression turn-on and turn-off during cell state transitions at single-cell resolution. Gene expression data were binarized to a 1 (on) or 0 (off) state using the binarize_exp function (fix_cutoff = TRUE, binarize_cutoff = 0.05) from the GeneSwitches package. A mixture model of two Gaussian distributions was fitted to the input gene expression for each gene, which was used to calculate a threshold for binarizing the gene. Genes without a significant "on–off" bimodal distribution were removed, and the binary state of gene expression (on or off) was modeled using the find_switch_logistic_fastglm function (downsample = TRUE). The top 50 best-fit (high McFadden's Pseudo R^2^) genes were plotted along the proposed timeline. Genes turned on with the proposed time were shown above the horizontal axis. Genes that were turned off with the proposed time are shown below the horizontal axis.

### Histological observation

#### Hematoxylin and eosin staining

The ovary samples were fixed in 4% polyformaldehyde, embedded in paraffin, sectioned, and stained with hematoxylin and eosin (H&E) for the histologically observation ovarian tissues. SlideViewer 2.5.0 (3DHistech, Budapest, Hungary) was used for imaging.

#### Immunofluorescence staining

The sections of paraffin-embedded tissues were stained with Decorin antibody (sc-73896, Santa Cruz, Dallas, Texas, USA), VE-cadherin antibody (sc-9989, Santa Cruz, Dallas, Texas, USA), CD53 antibody (sc-9989, Santa Cruz, Dallas, Texas, USA), FSHR antibody (22665-1-AP, Proteintech, Rosemont, Illinois, USA), RGS5 antibody (11590-1-AP, Rosemont, Illinois, USA), and DAPI (C0060, Solarbio, Beijing, China) for immunofluorescence. SlideViewer 2.5.0 (3DHistech, Budapest, Hungary) was used for imaging.

### Statistical analysis

The Student's *t*-test was used for blood biochemical and cell types proportion data analysis by SPSS software, version 24.0. (IBM, Armonk, New York, USA). Statistical significance was set at *P* < 0.05.

## Results

### Blood biochemical and body weight of sheep

Twelve hours before sampling the ovaries, the venous blood samples were collected to evaluate the physiological status of the sheep. As shown in Table 1, the blood biochemical and body weight of sheep had no difference between the two groups.

**Table 1:** Blood biochemical of sheep

Item	LLS	HLS	*P*-value
TP, g/L	70.70 ± 2.61	76.03 ± 2.41	0.923
GLU, mmol/L	3.73 ± 0.29	3.27 ± 0.17	0.339
TC, mmol/L	1.98 ± 0.27	1.57 ± 0.26	0.980
HDL-C, mmol/L	1.41 ± 0.44	0.99 ± 0.24	0.373
LDL-C, mmol/L	0.80 ± 0.10	0.65 ± 0.09	0.953
TG, mmol/L	0.17 ± 0.04	0.20 ± 0.05	0.571
E_2_, ng/L	58.50 ± 6.38	58.59 ± 1.49	0.060
LH, ng/L	17.75 ± 2.44	13.84 ± 0.57	0.210
FSH, IU/L	32.53 ± 2.98	29.93 ± 3.84	0.511
T, nmol/L	7.42 ± 0.19	5.86 ± 0.10	0.328
Prog, pmol/L	941.88 ± 46.44	1,057.59 ± 152.12	0.057

*E*_*2*_ Estradiol, *FSH* Follicle-stimulating hormone, *GLU* Glucose, *HDL-C* High-density lipoprotein cholesterol, *LDL-C* Low-density lipoprotein cholesterol, *LH* Luteinizing hormone, *Prog* Progesterone, *TC* Total cholesterol, *TG* Triglycerides, *T* Testosterone, *TP* Total protein

### Clustering and identification of the ovarian somatic cells

**Fig. 1:**
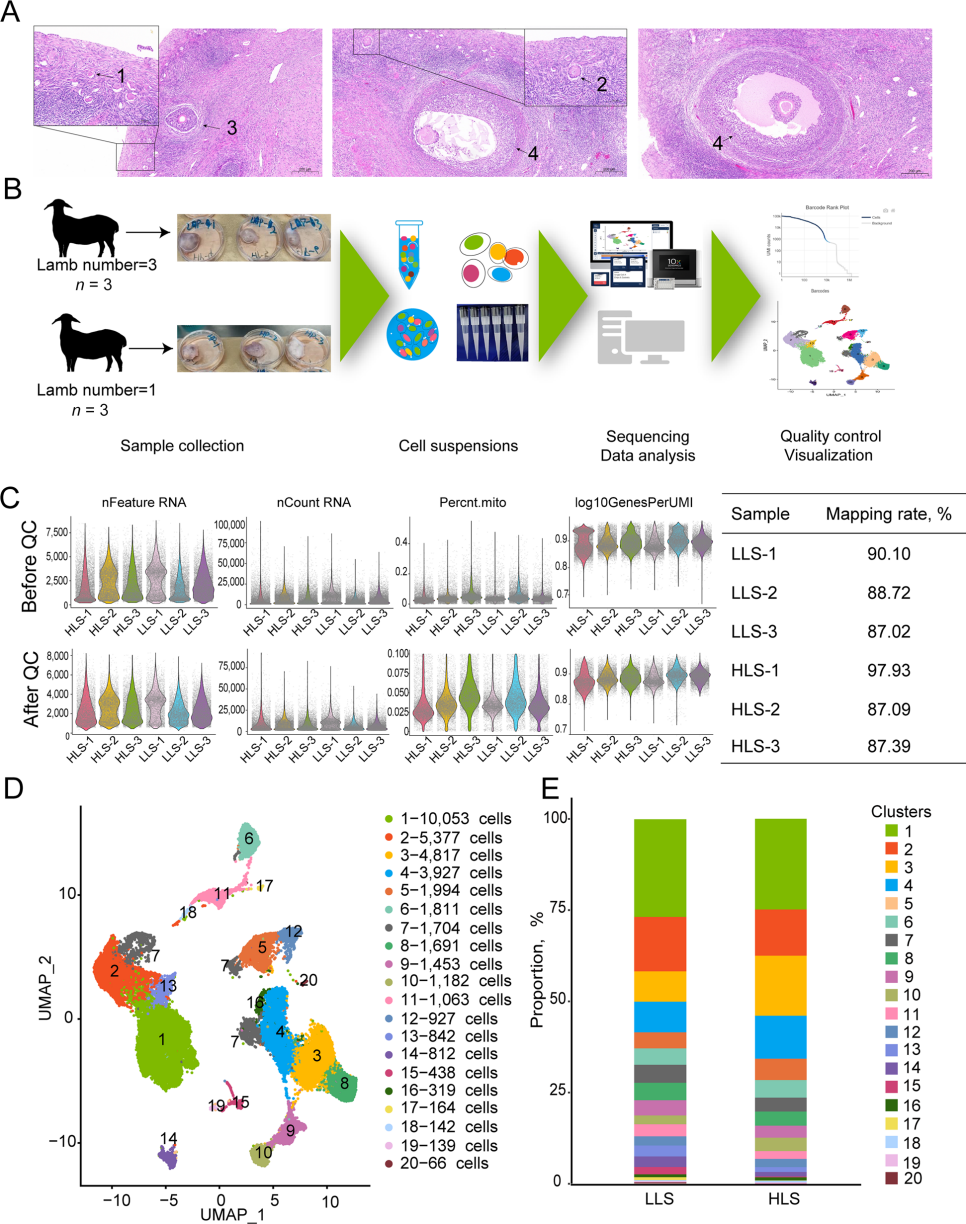
Single cell transcriptome sequencing of somatic cells in Hu sheep ovary. **A** H&E staining of the ovary; 1, Primordial follicle; 2, Growing follicle; 3, Antral follicle; 4, Graafian follicle. **B** Procedure of ovary single-cell transcription sequencing. **C** Quality control of single-cell transcriptome data. **D** UMAP of ovary single-cell transcription sequencing data and clusters distribution in HLS and LLS groups

In this study, ovaries were obtained from 6 ewes (3 replicates in each group) with different litter sizes, and subjected to H&E staining was performed for ovary structure observation. In the ovary, follicles were observed in different developmental states in both groups (Fig. 1A), which means that our subsequent analysis covers all cell types from various developmental states during estrus.

The ovaries were digested for 10× genomic scRNA-seq (Fig. 1B). After critical cell filtration, 38,921 cells were collected. The number of cells obtained from every sample ranged from 5,796 to 7,335, and the average number of UMI in each cell ranged from 6,227 to 10,384. The average number of genes in each cell ranged from 2,160 to 3,003, and the average proportion of mitochondrial UMI in each cell ranged from 0.0330 to 0.0480 (Fig. 1C). Mapping rate of every sample was higher than 85%, indicating the reliability of the scRNA-seq data of this study. Based on the sequencing data, a seurat-based workflow was used for cell clustering, and a total of 20 clusters (C) were identified by the uniform manifold approximation and projection (UMAP) analysis (Fig. 1D). All clusters were present in HLS and LLS groups (Fig. 1E).

**Fig. 2:**
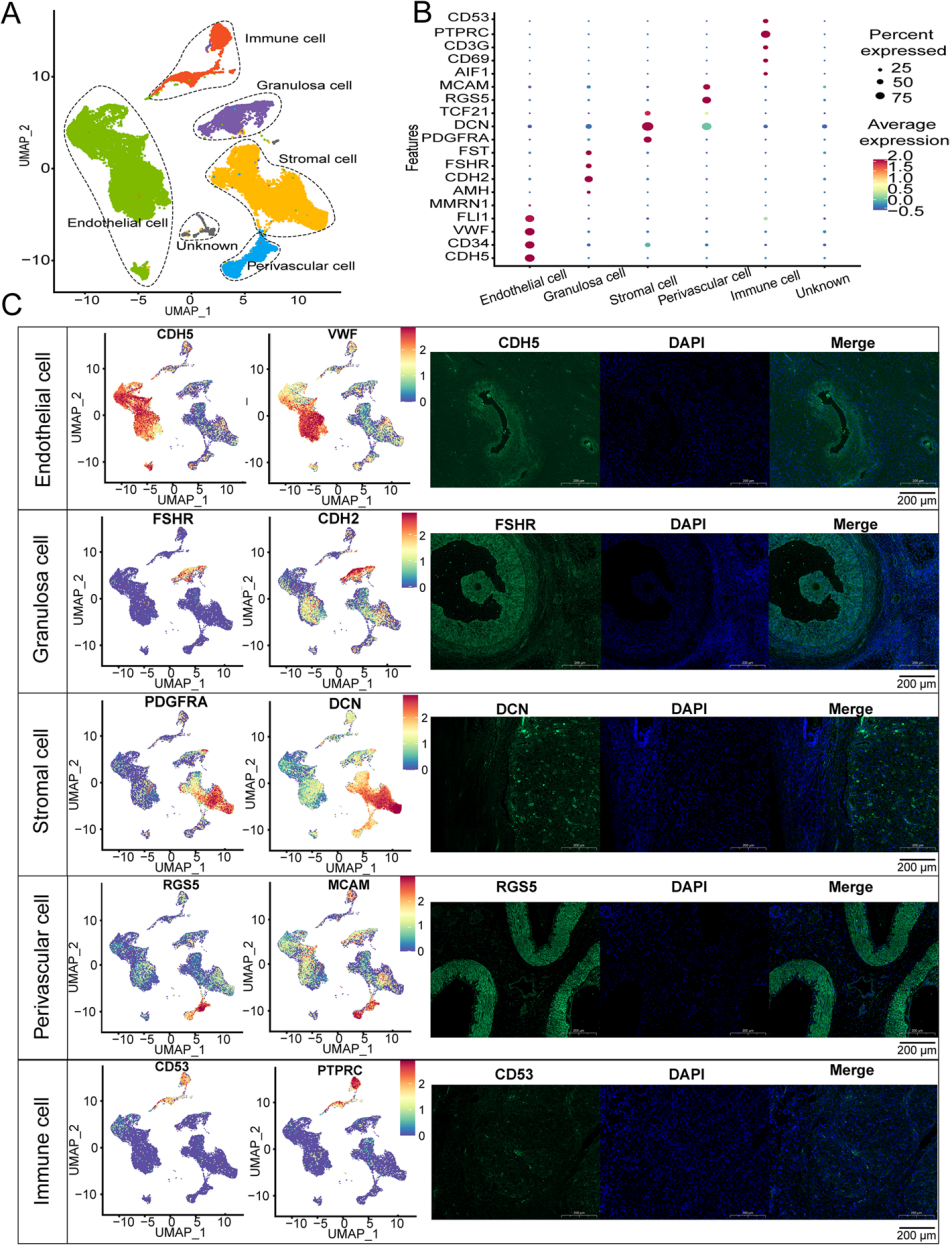
Identification of somatic cells in Hu sheep ovary. **A** Identification result of five different cell types on UMAP. **B** Dot plot of different cell marker gene expression levels. **C** Representative marker genes’ feature plot and immunofluorescence of ovary somatic cell, green indicates gene, and blue indicates DAPI. Scale bar: 200 μm

We characterized ovarian somatic cell types by existing cell markers provided by reference. The cells in C1, C2, C7-1, C13, and C14 were endothelial cells with high expression levels of marker genes, including *CDH5*, *CD34*, *VWF*, *FLI1*, and *MMRN1* [40]. Granulosa cells (GCs) (C5, C7-2, and C12) were recognized by the expression levels of *AMH*, *CDH2*, *FSHR*, and *FST* [41, 42]. According to the high expression levels of *PDGFRA, DCN*, and *TCF21*, cells in C3, C4, C7-3, C8, and C16 were identified as ovarian stromal cells [43]. Cells in C9 and C10 were annotated as perivascular cells based on the high expression of typical cell markers *RGS5, MCAM,* and *DES* [44]. The immune cells (C6, C11, C17, and C18) recognized by expression levels of *CD69*, *CD3G*, *PTPRC* and *CD53* [41]. The gene signatures of cells in C15, C19, and C20 were *DCDC2*, *MUC16*, *MPZ*, *CDH19*, *MZB1*, and *VPREB3.* The specific cell type related to these markers is rarely reported. Thus, these cells were identified as “unknown cells” (Fig. 2A). The immunofluorescence results also confirmed the expression and localization of different cell types (Fig. 2B and C). After cell type identification, five cell types of ovarian somatic from Hu-sheep were identified, and the cell number of endothelial cells, GCs, stromal cells, perivascular cells, and immune cells were 17,921, 3,214, 11,328, 2,635, and 3,180, respectively.

### Ovarian somatic cell expression profiles during estrus

**Fig. 3:**
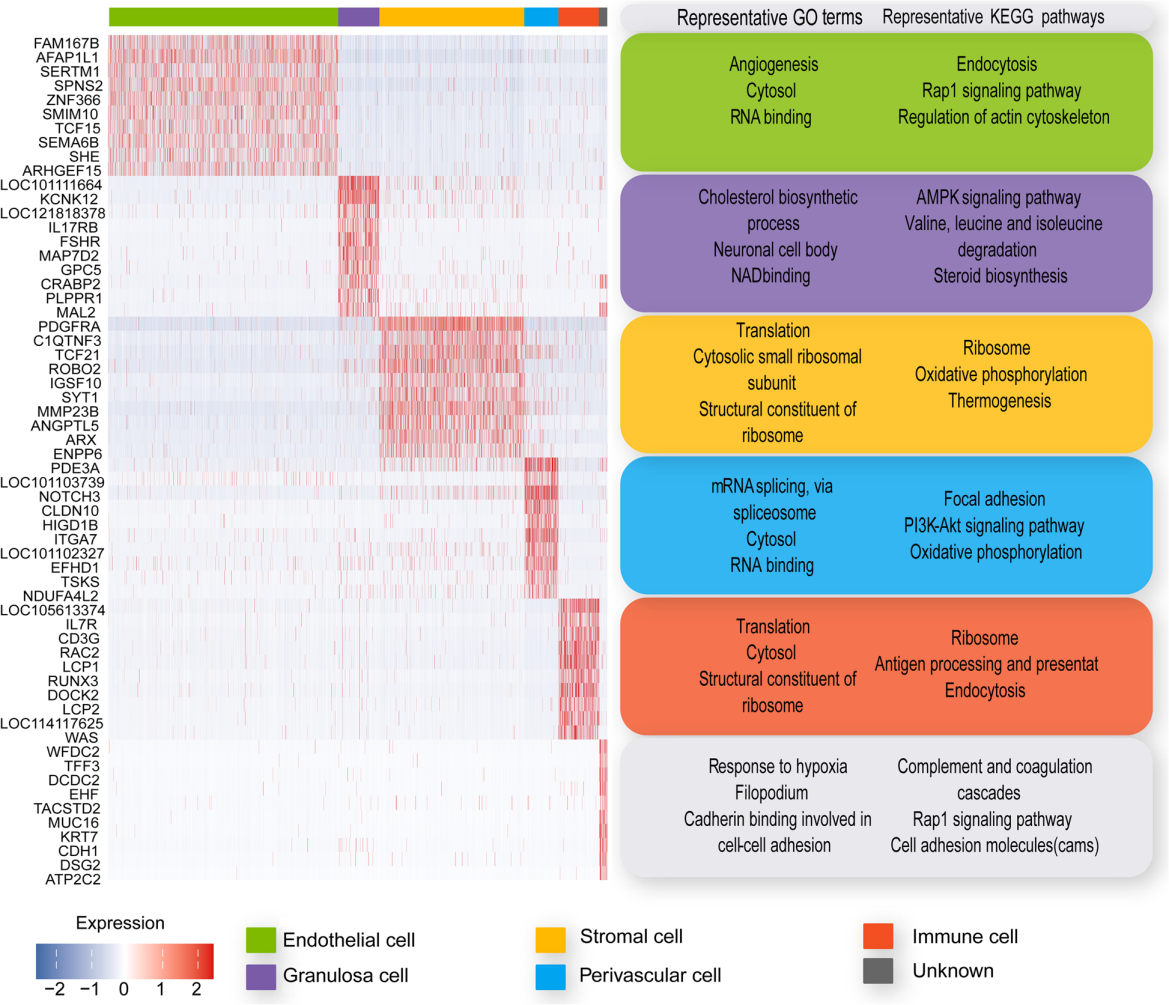
Ovary somatic cells marker genes heatmap and function enrichment

We collected high-variable genes (Top 300) of each cell type for GO and KEGG enrichment analysis. The top 3 GO terms and KEGG pathways are shown in Fig. 3. GO and KEGG enrichment data uncovered the typical cell function and confirmed the cell type identification results. Numerous pathways related to metabolism (energy, amino acid, carbohydrate, and lipid) occurred in GCs and stromal cells, which was indicated during estrus. GCs and stromal cells underwent a very energetic metabolism for follicle development and maturation. Some key pathways were enriched in certain cells during estrus. The “AMP-activated protein kinase (AMPK)” pathway is only enriched in GCs. “Forkhead box O (FoXo) signaling” pathway is enriched in GCs and stromal cells. “Phosphoinositide-3 kinase (PI3K)-Akt signaling” pathway enriched in endothelial and perivascular cells. The “Mitogen-activated protein kinase (MAPK) signaling” pathway enriched in endothelial and immune cells.

### Differences of ovarian somatic cell expression profiles between the HLS and LLS groups

**Fig. 4:**
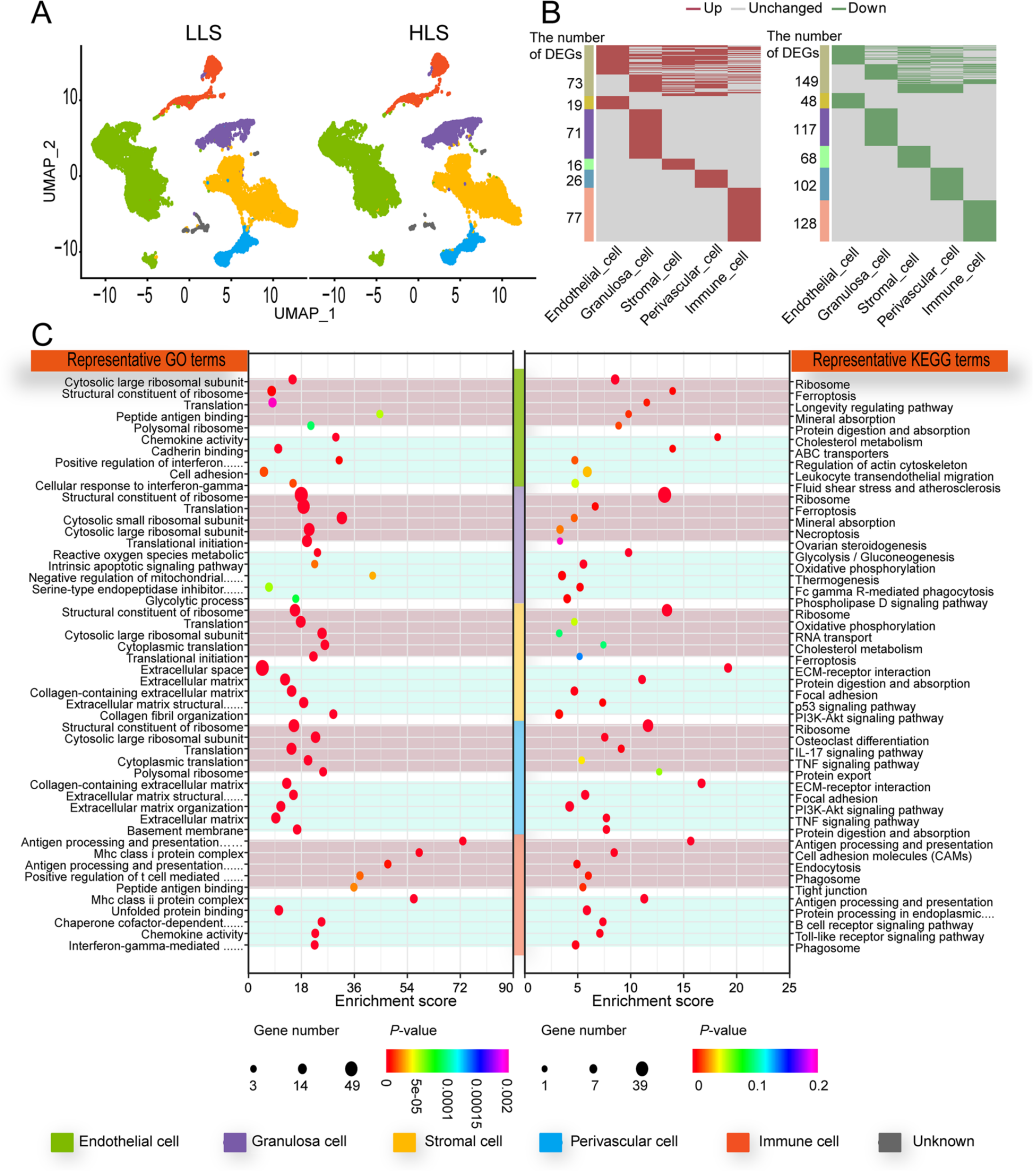
Comparison of differed cell type expression profiles between single and multi lamb sheep ovary. **A** Somatic cell type difference in UMAP. **B** Differentially expressed genes (DEGs) in a somatic cell of sheep ovary. **C** Top 5 Gene Ontology (GO) and Kyoto Encyclopedia of Genes and Genomes (KEGG) enrichment, light red indicates up-regulated, light blue indicates down-regulated

The differences between the somatic cell expression profiles of primiparous and multiparous Hu ewes were compared. In our study, the comparison of somatic cell expression profiles was based on the cell types. The proportion of cell types differed between the HLS and LLS groups. The proportion of endothelial and immune cells in HLS was lower than in LLS, whereas GCs and stromal cells were higher than in LLS. The distribution of perivascular cells was consistent in both groups (Table 2, Fig. 4A).

**Table 2:** Comparison of different cell type proportions between HLS and LLS

Cell type	LLS	HLS	*P*-value
Endothelial cell	49.95 ± 8.44	41.09 ± 8.63	0.86
Granulosa cell	7.63 ± 1.86	9.09 ± 3.23	0.27
Stromal cell	23.89 ± 4.52	34.74 ± 3.55	0.61
Perivascular cell	6.59 ± 0.48	6.88 ± 0.67	0.54
Immune cell	9.1 ± 1.74	7.53 ± 3.02	0.29
Unknown	2.84 ± 1.88	0.67 ± 0.09	0.02

*P*-value < 0.05 and fold change > 1.2 were used as screening criteria. The numbers of up-regulated DEGs were 61, 115, 65, 74, and 105, while the down-regulated genes were 179, 168, 108, 181, and 179 in endothelial cells, GCs, stromal cells, perivascular cells, and immune cells, respectively (Fig. 4B). GO and KEGG enrichment analyses were conducted with the identified DEGs (Fig. 4C). The enrichment results showed that the functions up-regulated in ovarian somatic cells were associated with ribosome in HLS group compared to LLS group. Up-regulation of structural components of ribosomes, cytoplasmic large ribosomal subunits, translation, and other GO terms were observed in endothelial, GC, stromal, and perivascular cells. The result of KEGG enrichment in ovarian somatic cells were closely involved in cellular functions and the enhanced ribosome pathways. The “ovarian steroidogenesis” pathway was up-regulated in GCs. Enhanced enrichment of the “oxidative phosphorylation” pathway was observed in stromal cells. Decreased functional enrichment of somatic cells was associated with extracellular structures and cell adhesion functions. For the GO term, cell adhesion enrichment was decreased in endothelial cells. Functional enrichment was decreased in stromal and perivascular cells. In KEGG enrichment, a corresponding decrease in functional enrichment (ECM-receptor interaction) was observed in stromal and perivascular cells. The HLS group showed decreased nutrient metabolic functions in the KEGG enrichment results compared to the LLS group, such as decreased “cholesterol metabolism” enrichment in endothelial cells and decreased enrichment of “protein digestion and absorption” in stromal and perivascular cells. This difference was more pronounced in GCs, where the HLS group significantly down-regulated enrichment of “glycolysis/glucose production”, “oxidative phosphorylation”, and “thermogenesis”. By comparing the expression profiles of ovarian somatic cells of Hu ewes with different litter size, the number of differential genes and functional changes closely related to ovarian ovulation was greater in GCs than in other somatic cells. Thus, the difference in GCs in the different groups required further analysis.

### GC subtype identification and developmental trajectory of sheep ovary

**Fig. 5:**
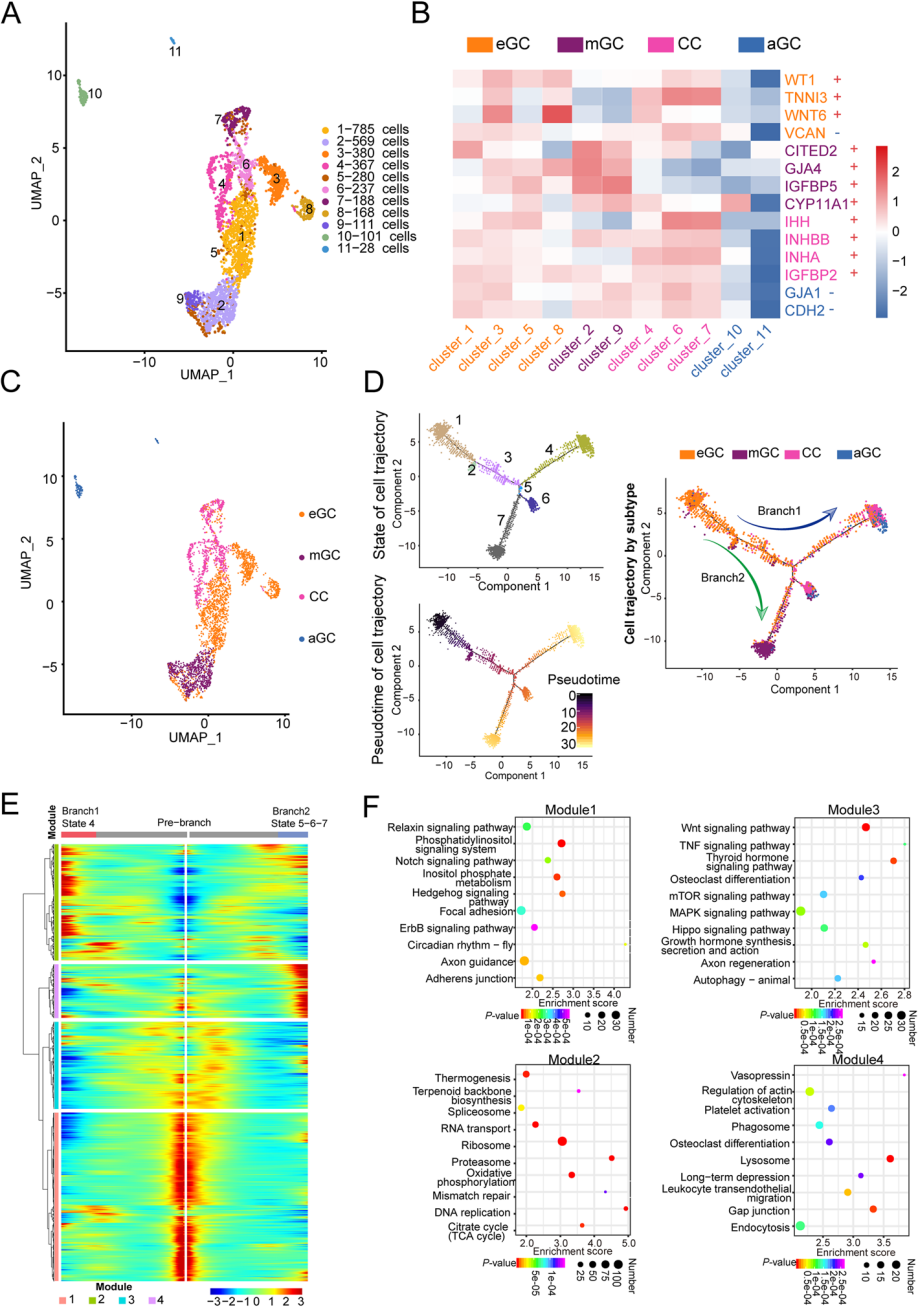
Granulosa cell subtype identification of sheep ovary. **A** UMAP of reduced the dimension of granulosa cells. **B** Granulosa cells sub-cluster marker gene heatmap. “-” indicates low expression of the corresponding gene. “ + ” indicates high expression of the corresponding gene. **C** Granulosa cell subtype identification in UMAP. **D** Granulosa cell pseudotime trajectory. **E** Granulosa cell different developmental states heatmap. **F** Top 10 KEGG enrichment of pseudotime heatmap genes

We reduced the dimension of the identified GCs into 11 sub-clusters (CL1–CL11) (Fig. 5A). CL1, CL3, CL5, and CL8 were recognized as early GC (eGC) through high expression of *WT1 *[45], *TNNI3* [41] and *WNT6* [46], and the low expression of *VCAN* [47]. Although we recognized these cells, the mapping condition was not ideal. Thus, we performed cell functions analysis with GO and KEGG (Fig. S1). Based on the GO and KEGG enrichment results, we found that the enriched GO terms and KEGG pathways related to signal transduction, response to peptide hormone and insulin, and the key pathways, such as “Rap1 signaling”, “FoxO signaling”, “AMPK signaling”, “Mammalian target of rapamycin (mTOR) signaling”, and “WNT signaling” pathways enriched in CL1. Positive regulation of transcription by RNA polymerase II, nucleus, and DNA binding function was enriched in CL3. “MAPK signaling”, “steroid biosynthesis”, “focal adhesion”, “PI3K-Akt signaling”, and “SMAD binding” pathways were enriched in CL3. In CL5, the enriched GO terms and KEGG pathways related to the nucleus and RNA binding were enriched. The enriched pathways were the “regulation of the actin cytoskeleton”, “focal adhesion”, and “relaxin signaling”. GO terms related to the nucleus, transcription corepressor activity, transcription factor binding and response to cAMP enriched in CL8, and pathways about “PI3K-Akt signaling” and “MAPK signaling” were enriched. Previous studies have shown that in in that the WNT signal activation occurs exclusively at the primordial follicle stage [46]. Meanwhile, the “FoXO signaling”, “mTOR signaling”, “MAPK signaling”, and “PI3K-Akt signaling” pathways have key functions in the activation of primordial follicles [48, 49]. Through the function enrichment results, we confirmed that these cell clusters belonged to eGC. Mural GCs (mGCs) (CL2 and CL9) were identified based on the expression levels of reported cell markers *CITED2* [50], *FSHR* [51], *GJA4, IGFBP5,* and *CYP11A1* [52, 53]*.* CL4, CL6, and CL7 were cumulus GCs (CCs) since the high expression levels of marker genes *IHH*, *INHBB*, and *IGFBP2* [43], while CL10 and CL11 were recognized as atretic GCs (aGCs). The expression levels of *GJA1* and *CDH2* were lower in atretic follicles GC when compared with healthy follicles.

We found a very extensive co-expression occurring in different GCs, which indicated that a lot of cell differentiation took place during estrus. Although the ovaries were collected at a single time point, the special histology structure of the ovary could obtain follicles in different developmental states (Fig. 1A). Cell trajectory analysis explored the differentiation trajectory of GCs by Monocle [38]. From trajectory analysis data, GC cells were divided into seven states. All CLs were presented in the trajectory. CL1 was presented almost in all states. CL2 was presented in states 6 and 7. CL3 was presented in states 1, 2, and 3. CL4 was presented in state 4. CL5 was presented in states 1 and 4. CL6 was presented in state 6. CL7 was presented in states 6 and 7. CL8 was presented in states 1 and 4. CL9 was presented in state 7, and CL10 was presented in state 4 (Fig. S2). We identified the GCs’ sub-type and mapped the sub-type into the trajectory. The identified sub-type and the eGCs were located in the early state. The GCs were differentiated into two broad directions, including mGCs and CCs. Through trajectory analysis, in eGCs, CL1 appeared in all states, and CL3 and CL8 appeared only in early development states, whereas CL5 intended to differentiate into CC (Fig. 5D). We performed heatmap analysis on cells in different developmental states and granulosa cells exhibited four patterns of gene expression levels. Genes in the pre-branch showed model1 and model3 patterns, with high expression in the early developmental stages. Genes in branch1 and branch2 showed similar patterns of late high expression levels in the two different branches (Fig. 5E). KEGG and GO enrichment analyses of genes were conducted with different expression patterns, and the enrichment results of model1 and model3 were similar to those of eGC enriched in “adherens junction”, “notch signaling”, “WNT signaling”, “thyroid hormone signaling”, “MAPK signaling”, “Hippo signaling”, and “mTOR signaling” pathways. The growth hormone synthesis, secretion, and action pathways were enriched in model1, indicating that cells were in a rapid growth period during this stage. In model2, significant enrichment of functions was related to genetic material transfer, such as the ribosome, proteasome, DNA replication, RNA transport, and mismatch repair, as well as enhanced cell metabolism activities such as oxidative phosphorylation, thermogenesis, and citrate cycle, which suggests that cell metabolism is activated during the process of early GCs to cumulus cells development. In the enrichment results of model4, functions related to the regulation of actin cytoskeleton, endocytosis, and phagosome were observed (Fig. 5F). These results justify the GC subtype identification strategy. The marker genes of each GC subtype were explored (Additional file 2). The maker genes of eGC, CC, and mGC identified in this study in Hu ewes were *WT1* and *CD34*, *AMH* and *INHA*, and *HTRA3*, respectively, for Hu sheep in our study.

**Fig. 6:**
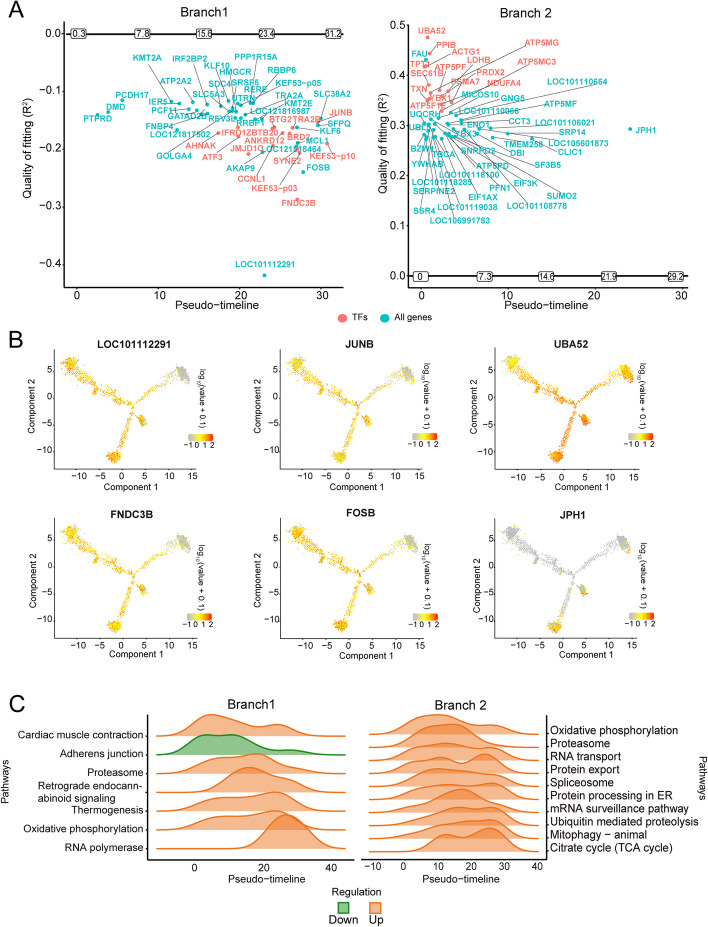
Granulosa cell developmental trajectory with pseudotime. **A** Key genes involved in the developmental process of granulosa cells. The horizontal axis is pseudotime, and the vertical axis is the goodness-of-fit R^2^. The genes turned on with the pseudotime are shown above the horizontal axis, and the genes turned off are shown below the vertical axis. Genes that satisfy the following conditions have been selected for mapping: 1. The percentage of zero-expressing cells is below 90%, 2. The top 50 plots with the highest goodness-of-fit. **B** feature plot of representative gene expression. **C** Top 10 KEGG enrichment of genes involved in granulosa cells developmental process. Up, function enrichment turned on; down, function enrichment closure

To investigate key genes involved in the developmental timeline of granulosa cells, GeneSwitches analysis was conducted. Along the timeline of the process of GCs developing to CCs (branch1), we observed gene closure throughout the entire timeline, leading to the development of early GCs into CCs. In the later stage, more transcription factors (TF), such as *JUNB* and *FNDC3B*, and a key gene, such as *FOSB*, participated in the closure process. At 23.4 h, *LOC101112291* expression arrested. These genes decreased expression levels during the final transformation into CCs (Fig. 6A and B). Along the timeline of GCs to mGCs development (branch2), the expression levels of genes increased in the early stage. Some TFs related to energy metabolisms, such as *ACTG1, LDHB, ATP5MG, ATP5MC3*, and *ATP5F1E*, expression initiated. In the late stage of the timeline, we observed the initiating of *JPH1* expression. These genes were expressed higher when early GCs were transformed into mGCs (Fig. 6A and B).

We performed KEGG pathway enrichment analysis on key genes in different branches. In the process of branch1, only "adherens junction" pathway was suspended. The other pathways were turned on. “Adherens junction” pathway was enriched at the lower levels in the early stages of development and decreased with the timeline, while “proteasome”, “thermogenesis”, and “oxidative phosphorylation” increased with the timeline and decreased rapidly before the endpoint. “RNA polymerase function” only increased in the late stages of development. In the process of branch2 development, the enrichment of pathways was turned on, focusing on cell energy metabolism (“oxidative phosphorylation” and “TCA cycle”) and related functions of cell protein synthesis (“RNA transport”, “protein export”, and “protein processing in the endoplasmic reticulum”) (Fig. 6C).

### Differences of ovarian GC expression profiles between the HLS and LLS group

**Fig. 7:**
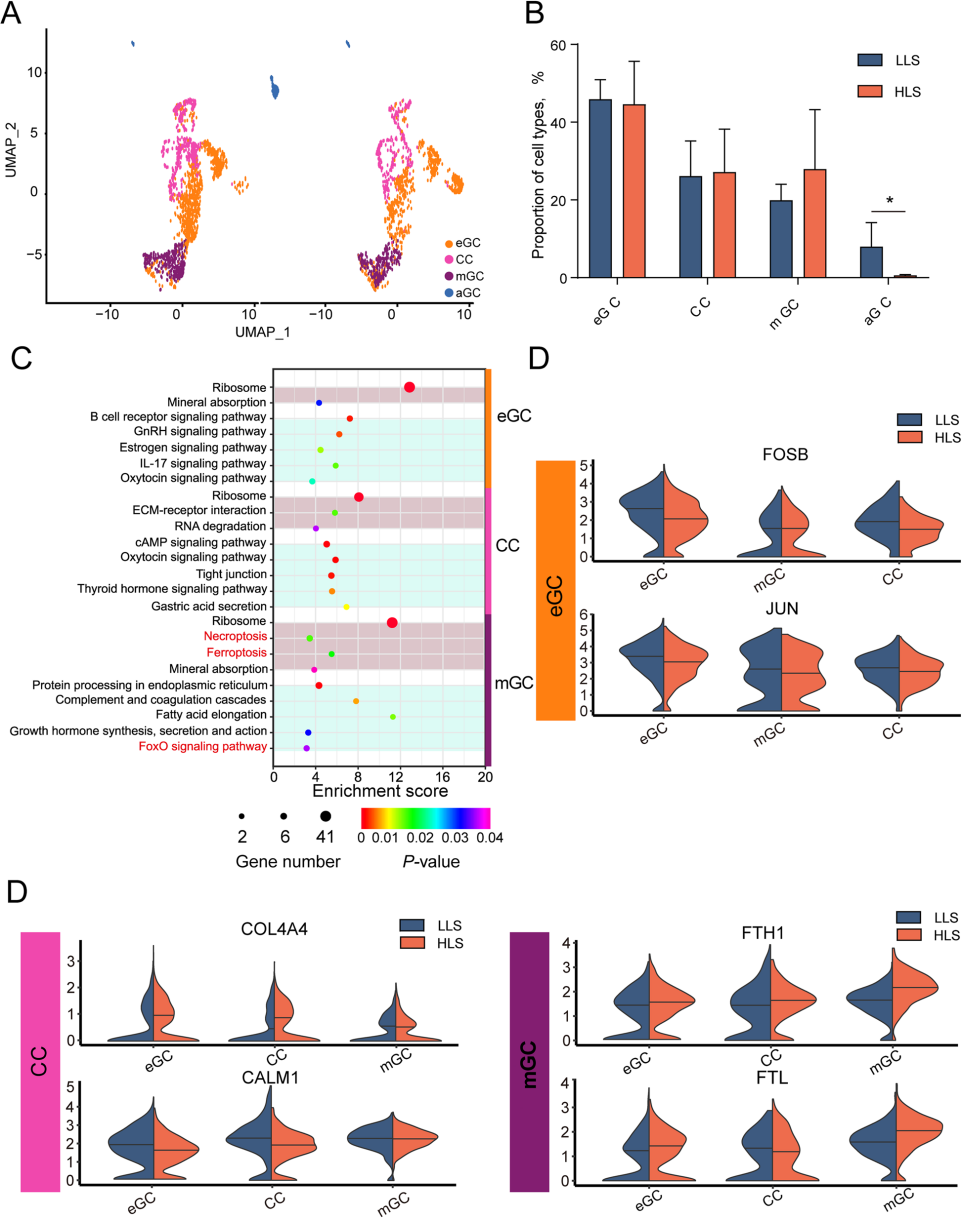
Comparison of different granulosa cell sub-type expression profiles between single/mult lamb sheep ovary. **A** Granulosa cell sub-type difference in UMAP. **B** Comparison of different granulosa cell sub-types proportion between HLS and LLS of cell Differentially expressed genes (DEGs) in a somatic cell of sheep ovary. **C** KEGG enrichment of DEGs in ovarian granulosa cell subtypes of sheep with different lambing numbers, light red indicates up-regulated, light blue indicates down-regulated. **D** Comparison of representative genes of ovarian granulosa cell subtypes in sheep with different lambing numbers

By comparing the proportions of different cell subtypes, the proportion of aGCs was significantly higher in the LLS group than in HLS (Fig. 7A). In the LLS group, the physiological status of GCs was altered, resulting in an increase in follicles with a propensity for atresia. Changes in cell function based on cell subtypes were analyzed using KEGG enrichment. Pathways associated with apoptosis and necroptosis were inhibited, while pathways associated with cell survival were up-regulated. This change was cell subtype based. In mGCs, the enrichment of the necroptosis pathway was elevated. The genes enriched in the pathway were *FTH1*, *FTL*, and *H2AZ1*. *FTH1* and *FTL* in the necroptosis pathway had a negative feedback regulatory effect in the ferroptosis pathway. In the current study, *FTH1* and *FTL* highly expressed in group HLS reduced necroptosis by reducing the release of reactive oxygen species (ROS) after decreased lysosome membrane permeabilization. The expression levels of ferroptosis-resisted genes *FTH1* and *FTL* were up-regulated in HLS. Then, we retrieved the location of *FTH1* and *FTL* on the ferroptosis pathway and analyzed its upstream and downstream genes. The downstream expression levels of key genes *MAP1LC3A*, *ATG5*, and *ATG7* (Fig. S3), and *NCOA4* (Fig. S3) were downregulated in the HLS group, thus reducing ferroptosis via inhibiting the Fenton response. On the other hand, the enrichment of the FoxO signaling pathway was downregulated in HLS and reduced apoptosis by decreasing the expression of *IRS2, EP300*, *BCL2L11*, and *SGK1* (Fig. S3). In CCs, the enrichment of ECM-receptor interaction in the HLS group was up-regulated by increasing the expression levels of *COL4A4* (Fig. 7D)*,* whereas the enrichment of the cAMP signaling pathway, oxytocin signaling pathway, tight junction, and thyroid hormone signaling pathway was down-regulated in the multi-lamb group, with decreasing the expression levels of *CALM1*, *PLD1*, *OXT*, *PLN*, *ATP2B1*, *F2R*, *ACTG1*, and *MYL6* (Fig. 7D, Fig. S3). In eGCs, differences between the two groups were reflected in the down-regulation of the expression of transcription factor complex AP-1 composed of *FOS*, *FOSB*, and *JUN* (Fig. 7B and C).

## Discussion

Every estrus cycle in sheep typically consists of three or four follicle waves development during the inter-ovulatory interval [54], and about 1–3 mature follicles ovulate [55]. Estrus is a special time window. In peripheral blood, luteinizing hormone peaks can be observed; estradiol decreases rapidly from maximal values; progesterone is at its lowest level, and ewe usually ovulate about 20 h after the onset of estrus [56]. Thus, understanding the transcriptional profiles of ovarian somatic cells during estrus is essential to investigate the mechanism of ovulation. In the present study, we investigated the differences in expression profiles of primiparous and multiparous Hu ewe ovary somatic cells by scRNA-seq, providing insight into the mechanisms underlying the differences in ovulation numbers. A total of five types of somatic cells were identified, and corresponding expression profiles were mapped in the ovaries of Hu ewe. A subtype identification of GCs was performed. Key genes involved in different subtype transitions were analyzed. The differences in cellular expression profiles were compared to identify the key factors regulating different litter sizes. These findings provide a theoretical basis for breeding high-fertility sheep and propose new targets for molecular genetics-based selection.

### Identification of ovarian somatic cells

Various cells in the ovary act in synergy to enable ovarian function, whereas existing research has not paid much attention to the function of these somatic cells, except GCs. The ovarian stroma comprises mostly incompletely characterized stromal cells (e.g., fibroblast-like, spindle-shaped, and stromal cells) [57]. In recent years, the role of stromal cells in the ovary has been revisited, and studies have identified estrogen receptors α and β in the cytoplasm and nucleus of bovine stromal cells, unlike fibroblasts, these cells are oval cells with lipid droplets and vacuoles [58]. Progesterone receptor α has been identified in stromal cells of pregnant and postpartum rabbit ovaries [59]. In the present study, a large amount of energy metabolism occurred in the ovaries during estrus for supporting ovulation. There is an extensive blood supply to the ovary, which is involved in forming dominant follicles, and the endothelium can participate extensively in the angiogenic process. The importance of combined transplantation of ovarian endothelial cells with stromal cells when performing follicular transplantation in individuals with premature ovarian failure was demonstrated to ensure the formation of a well-vascularized and well-structured ovarian-like stroma [60]. A previous study proposed that perivascular cells were multipotent progenitors that contribute to granulosa, thecal, and pericyte cell lineages in the ovary, which supports folliculogenesis [23]. The main functions of immune cells in the ovary are defense, remodeling of ovarian structure, signaling, and ovarian aging [22, 61]. In the present study, marker genes of ovary somatic cells proposed were confirmed in former studies [44] and also available as stroma cell gene signatures of Hu ewe.

Among the ovarian somatic cells, the most well-studied are the GCs. The GC is a somatic cell surrounding the oocyte co-located with the oocyte in the same follicular microenvironment. Its function is limited to the secretion of gonadotropins to stimulate ovulation and includes follicular development. GCs secrete factors, including gonadal steroids, growth factors, and cytokines are critical for GC survival and follicular growth [62, 63]. In contrast, identifying the GC subtype remains controversial, especially for sheep. In the human ovary, the expression pattern of early-stage GCs is *WT1*^high^/*EGR4*^high^/*VCAN*^low^/*FST*^low^. The expression pattern of CCs is (*VCAN*^high^/*FST*^high^/*IGFBP2*^high^/*HTRA1*^high^/*INHBB*^high^/*IHH*^high^), and the expression pattern of mGC is *WT1*^low^/*EGR4*^low^/ *KRT18*^high^/*CITED2*^high^/*LIHP*^high^/*AKIRIN1*^high^ [43]. In domestic animals, the GCs of goats were identified based on developmental trajectory. *ASIP* and *ASPN* were highly expressed in early GCs, *INHA*, *INHBA, MFGE8*, and *HSD17B1* were highly expressed in GCs during the growth phase, and *IGFBP2*, *IGFBP5*, and *CYP11A1* were highly expressed during the growth phase of GCs [42]. However, the study did not give subtype classification markers based on cell function. The present study defined GC subtypes by combining existing marker genes and functional analysis of different sub-clusters. Using pseudotime analysis, the reliability of GC subtype identification was verified. It has been found that *WT1* and *CD34* are marker genes for eGCs. *AMH* and *INHA* are marker genes for CCs, and *HTRA3* is a marker gene for mGCs. These marker genes were applicable for identifying sheep GC subtypes.

Five somatic cell lineages were identified in sheep ovaries based on their gene expression signatures. GCs were further characterized into three subtypes, marker genes for each cell type are only expressed in specific “regions” in the UMAP figure and immunofluorescence profiling, which were consistent with the anatomy of the ovary [64]. These results illustrated the reliability of the scRNA-seq data from this study. However, no luteal cells were detected in our dataset, which is consistent with our previous study [40], which implies the degradation of luteal cells during the samples collection period (estrus) or the luteal cells are difficult to collect.

### The transition of different GC subtypes

CC and mGC interact with oocytes differently in the follicle. The CC carries out bidirectional information transfer with the oocyte through gap junctions, contributing to oocyte maturation, fertilization, and early embryonic development [65]. In contrast, the mGC has multiple receptors on its surface that can secrete various hormones and cytokines that regulate follicular growth and maturation in an autocrine and paracrine manner [66]. In the present study, key genes were observed using GeneSwitches, which are involved in the transition of different GC subtypes. The suspend expression of *LOC101112291* led to the differentiation of eGCs into CCs, while the initiating of *JPH1* expression led to the differentiation of eGCs into mGCs. A previous study investigating the molecular mechanism of lambing in Hanper sheep using ovarian tissue has revealed that *LOC101112291* (*XIST*) regulates lambing number through the methylation process [20]. On the other hand, the protein expressed by the *JPH1* gene, junctophilins (JPHs), is a family of structural proteins that connect the plasma membrane with intracellular organelles such as the endoplasmic/sarcoplasmic reticulum (ER/SR). The anchoring of these membrane structures leads to highly organized subcellular connections, playing an important role in signal transduction in all excitable cell types [67]. Our study found that the expression levels of these genes expression were turned off. Therefore, *LOC101112291* and *JPH1* genes may potentially regulate the direction of differentiation of early GCs.

### Differences in transcriptional profiles of GCs in Hu sheep with different litter size

In the modern sheep production system, the reproductive performance of female animals determines the economic profitability of farming, and how to increase the number of lambs has always been the hottest spot and key in sheep breeding and reproduction research. Based on former studies, GC is vital in follicle development [62, 63, 68]. Our data revealed the number of differential genes and the key functional differences in primiparous and multiparous Hu ewes distributed in GCs, so we paid attention to these cell clusters. Li et al. [42] studied the gene expression of GCs at different stages in two populations of Jining Gray goats, and they found differences in the enrichment of GO terms of GCs at different periods in different litter size groups. The previous study showed the differences in the expression profiles of GCs at different litter size from functional analysis. In this study, the definition of the subtypes of Hu ewe GCs enabled us to discover differences in the functions of GCs in the two groups. Follicular atresia was increased in the LLS group, which was mainly caused by ferroptosis of GCs. Healthy growing follicles have a granulosa layer that is aligned with the follicular basement membrane, and no apoptotic cells are present. In the early stages of follicular atresia, apoptotic GCs gradually increase. In progressive atretic follicles, most GCs undergo apoptosis leading to severe disruption of the granulosa layer and clearance of the follicle. Apoptosis is initiated in the GCs on the inner surface of the granulosa layer, while the oocytes, as well as the inner and outer layers of the membrane, are not affected by apoptosis in the early stages of atresia [69], suggesting that GC apoptosis plays an initiating role in follicular atresia [70, 71]. Ferroptosis is a form of cell death caused by iron-dependent lipid peroxidation and ROS accumulation characterized by the reduction or loss of mitochondrial cristae and rupture of the outer mitochondrial and mitochondrial membranes condensation [72]. Zhang et al. [73] found that transferrin expression was significantly reduced, and *PCBP* expression was significantly increased in porcine early atretic follicles, suggesting that iron accumulation began to occur early in follicular atresia and ferroptosis had an essential regulatory role in follicular atresia. Another study on female infertility found that induced iron overload in GCs led to ferroptosis and suppressed oocyte maturation by releasing exosomes from GCs, suggesting that ferroptosis of GCs is detrimental to oocyte development [74]. This study found that the GCs of multiparous ewes suppressed ferroptosis by increasing the expression levels of anti-ferroptosis genes *FTH1* and *FTL*, which promotes oocyte maturation and prevents follicular atresia, contributing to the multiparous trait.

## Conclusion

In our study, we identified differences in the expression profiles of ovarian somatic cells between primiparous and multiparous Hu ewes. These differences were mainly attributed to GCs. The expression condition of *JPH1* and *LOC101112291* emerged as significant indicators for determining the evolutionary directions of granulosa cells. Additionally, *FTH* and *FTL* potentially regulate litter size by inhibiting granulosa cells ferroptosis and promoting follicle development*.* This study provides new insights into the molecular mechanisms underlying the high reproductive rate of Hu sheep.

### Supplementary Information

**Additional file 1: Table S1. **Sheep weight and production records. **Table S2.** Blood biochemical ELISA kit information. **Fig. S1.** Function enrichment of granule cell sub-cluster marker genes. **Fig. S2.** Cell trajectory by clusters. **Fig. S3.** The relative expression of key genes in the pathways in granulosa cell subtypes.

**Additional file 2. **Top 20 marker genes of GC subtype.

## Data Availability

The datasets generated and/or analysed during the current study are available in the [NCBI gene expression omnibus database] repository [https://www.ncbi.nlm.nih.gov/geo/query/acc.cgi?acc=GSE233801, Accession Number: GSE233801].

## Abbreviations

aGC: Atretic granulosa cell
AMPK: AMP-activated protein kinase
C: Clusters
CC: Cumulus granulosa cell
CL: Sub-cluster
DEG: Differentially expressed gene
eGC: Early granulosa cell
ER/SR: Endoplasmic/sarcoplasmic reticulum
FoXo: Forkhead box O
GC: Granulosa cell
GEM: Gel beads in emulsions
GO: Gene Ontology
H&E: Hematoxylin and eosin
HLS: Highly reproductive group
KEGG: Kyoto Encyclopaedia of Genes and Genomes
LLS: Lowly reproductive group
MAPK: Mitogen-activated protein kinase
mGC: Mural granulosa cell
mTOR: Mammalian target of rapamycin
PCA: Principal component analysis
PCR: Polymerase chain reaction
PI3K: Phosphatidylinositol 3-kinase B
ROS: Reactive oxygen species
scRNA-seq: Single-cell RNA sequencing
TF: Transcription factors
UMAP: Uniform Manifold Approximation and Projection
UMI: Unique molecular identifiers
