# Supplementary material for: Single-cell sequencing reveals the reproductive variations between primiparous and multiparous Hu ewes
Source: J Anim Sci Biotechnol. 2023 Nov 14;14:144. doi: 10.1186/s40104-023-00941-1 (PMC10644470; doi:10.1186/s40104-023-00941-1)
Supplement: Supplementary file 1 — Additional file 1: Table S1. Sheep weight and production records. Table S2. Blood biochemical ELISA kit information. Fig. S1. Function enrichment of granule cell sub-cluster marker genes. Fig. S2. Cell trajectory by clusters. Fig. S3. The relative expression of key genes in the pathways in granulosa cell subtypes. [file 40104_2023_941_MOESM1_ESM.docx]

**Table S1** Sheep weight and production records

| **Sheep** | **Weight, kg** | **Litter size** | **Birth date** | **Birth weight of lamb, kg** |
| --- | --- | --- | --- | --- |
| HLS-1 | 37.87 | 3 | 2020.4.5 | 2.6/2.8/2.6 |
|  |  |  | 2020.11.27 | 2.9/2.8/2.8 |
|  |  |  | 2021.8.31 | 2.4/2.8/2.7 |
| HLS-2 | 40.74 | 3 | 2020.4.11 | 2.3/2.7/2.36 |
|  |  |  | 2020.12.8 | 2.7/2.9/3.0 |
|  |  |  | 2021.9.8 | 3.0/2.9/2.7 |
| HLS-3 | 41.88 | 3 | 2020.3.30 | 3.0/2.8/2.9 |
|  |  |  | 2020.11.25 | 3.1/2.6/2.8 |
|  |  |  | 2021.8.28 | 2.8/3.0/2.8 |
| LLS-1 | 41.39 | 1 | 2020.4.15 | 4.3 |
|  |  |  | 2020.11.30 | 5 |
|  |  |  | 2021.8.28 | 5.2 |
| LLS-2 | 42.41 | 1 | 2020.4.7 | 3.9 |
|  |  |  | 2020.12.1 | 4.1 |
|  |  |  | 2021.9.5 | 3.8 |
| LLS-3 | 42.23 | 1 | 2020.4.15 | 4.6 |
|  |  |  | 2020.12.10 | 4.4 |
|  |  |  | 2021.9.01 | 4.3 |

**Table S2** Blood biochemical ELISA kit information

| **Item** | **Name of kit** | **Item number** | **Manufacturer** | **Origin** | |
| --- | --- | --- | --- | --- | --- |
| E_2_ | Ovine estradiol, E2 ELISA kit | F3906-B | Kexing Trading Co. Ltd. | Shanghai,  China |  |
| LH | Ovine luteinizing hormone, LH ELISA kit | F72141-B |  |  |  |
| FSH | Ovine follicle-stimulating hormone, FSH ELISA kit | F7100011-B |  |  |  |
| T | Ovine testosterone, T ELISA kit | F3907-B |  |  |  |
| Prog | Ovine progesterone, PROG ELISA kit | F3911-B |  |  |  |


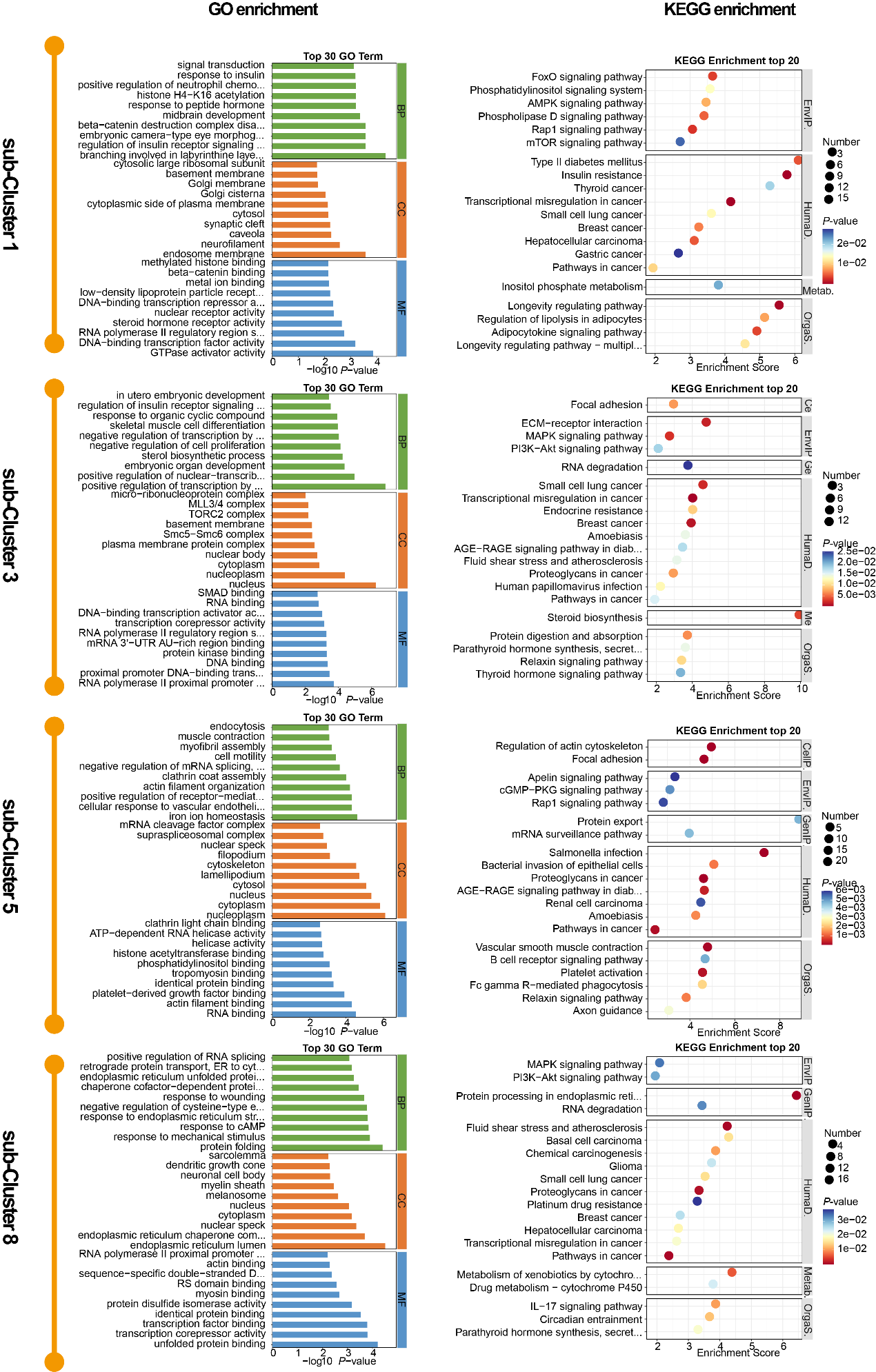


**Fig. S1** Function enrichment of granule cell sub-cluster marker genes


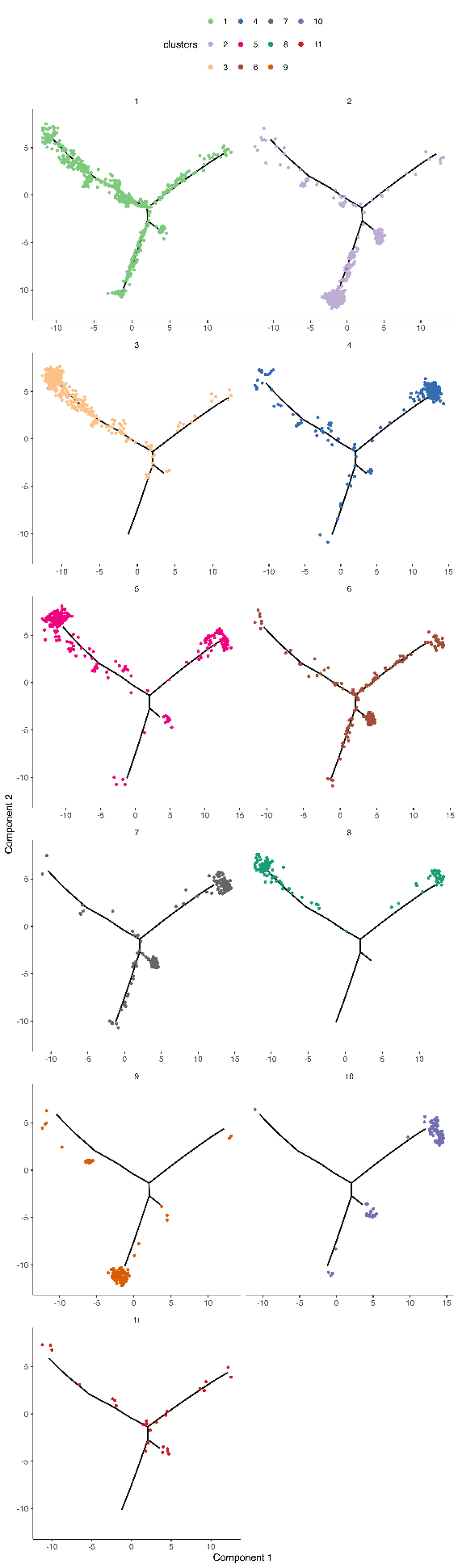


**Fig. S2** Cell trajectory by clusters


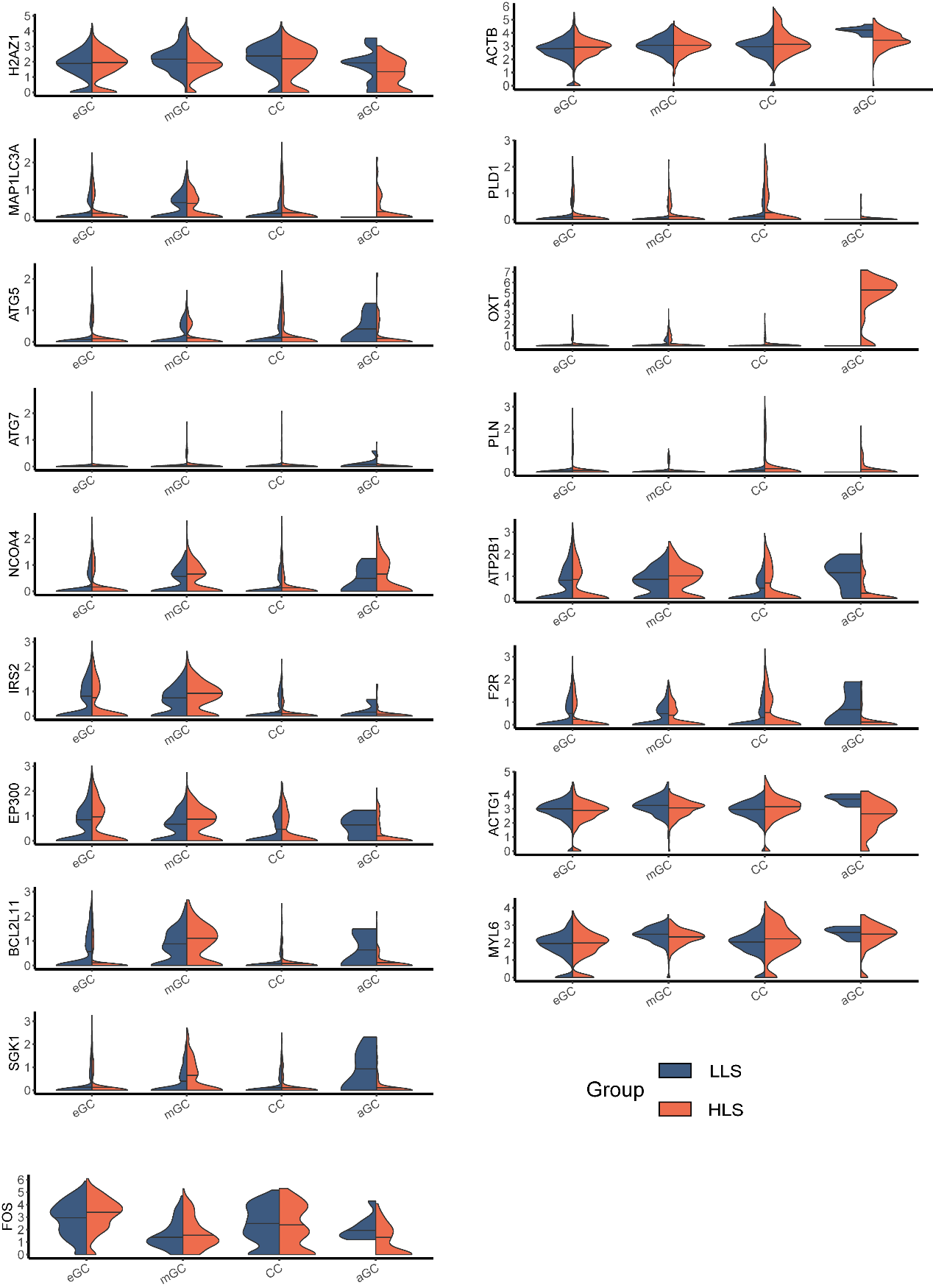


**Fig. S3** The relative expression of key genes in the pathways in granulosa cell subtypes
